# Supplementary material for: A RAF-SnRK2 kinase cascade mediates early osmotic stress signaling in higher plants
Source: Nat Commun. 2020 Jan 30;11:613. doi: 10.1038/s41467-020-14477-9 (PMC6992735; doi:10.1038/s41467-020-14477-9)
Supplement: Supplementary file 1 — Supplementary Information [file 41467_2020_14477_MOESM1_ESM.pdf]

A RAF-SnRK2 kinase cascade mediates early osmotic stress signaling in higher plants

Lin et al.

Supplementary Information

This file includes:

Supplementary Figure 1 to Figure 11 with figure legends and Supplementary Table 1.

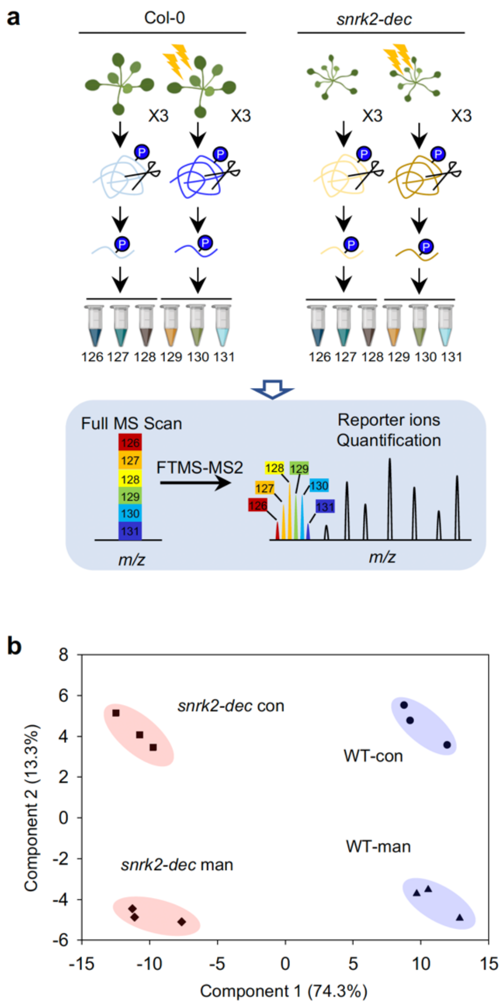

Supplementary Fig. 1 Quantitative phosphoproteomics reveal the osmotic stress responsive phosphoprofiles. **a** TMT6-plex phosphoproteome analysis was used to identify the protein kinases that have increased phosphorylation after 30 min of mannitol treatment of wild type and *snrk2-dec* mutant plants in biological triplicates. Proteins were extracted, precipitated, and digested using Lys-C followed by trypsin. The phosphopeptides were labeled with TMT6-plex reagents, pooled, and enriched using an IMAC StageTip. The enriched phosphopeptides were fractionated by a basic pH reverse phase StageTip. The fractions were analyzed by FTMS-MS2. **b** PCA of the phosphorylation sites across all four samples representing distinct phosphoproteomic types for different samples and treatments (FDR < 0.05).

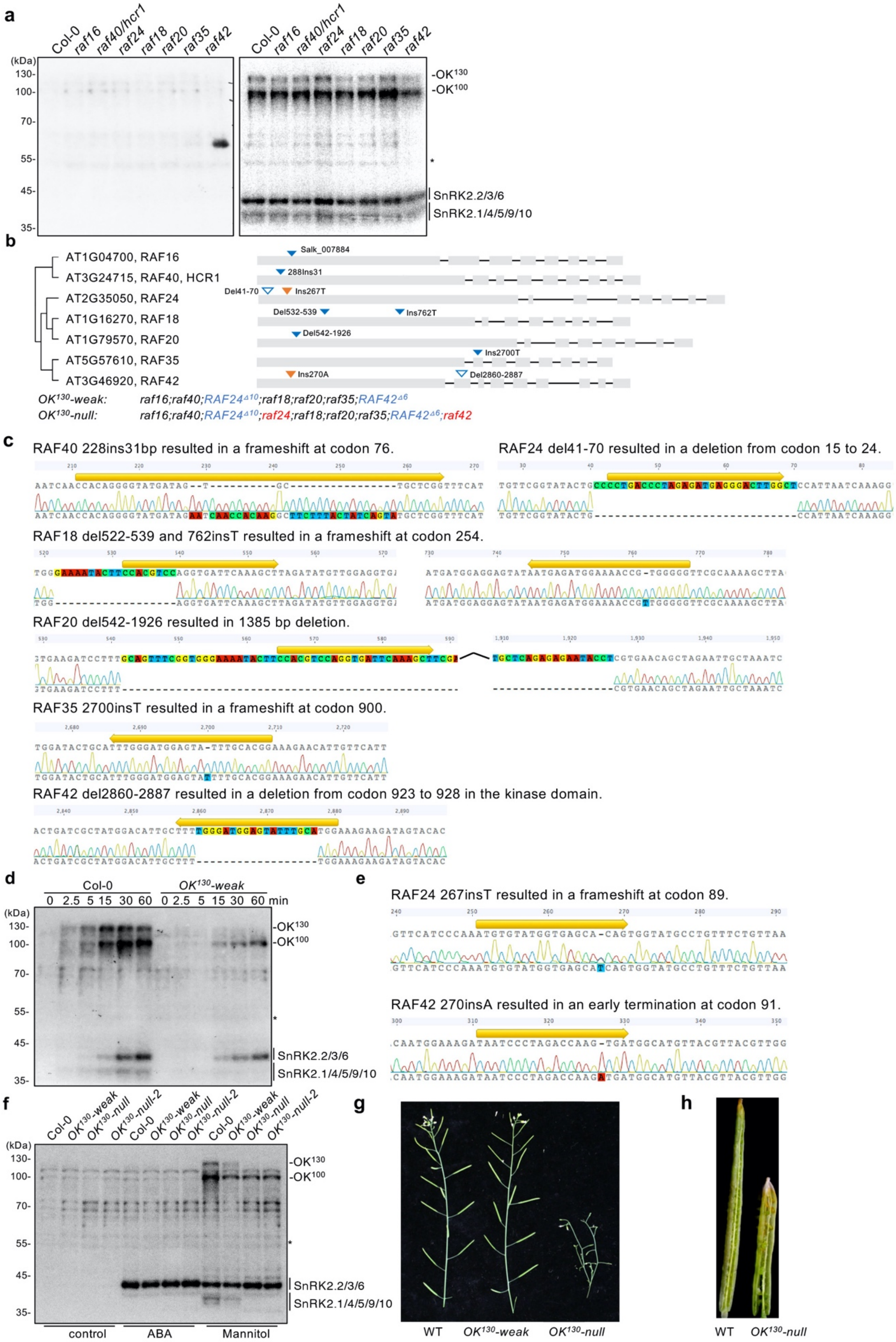
a
Col-0
raf16
raf40/hcr1
raf24
raf18
raf20
raf35
raf42
(kDa)
130-
100-
70-
55-
45-
35-
-OK130
-OK100
*
SnRK2.2/3/6
SnRK2.1/4/5/9/10
b
AT1G04700, RAF16
AT3G24715, RAF40, HCR1
AT2G35050, RAF24
AT1G16270, RAF18
AT1G79570, RAF20
AT5G57610, RAF35
AT3G46920, RAF42
Salk_007884
288Ins31
Del41-70
Ins267T
Del532-539
Ins762T
Del542-1926
Ins2700T
Ins270A
Del2860-2887
OK130-weak: raf16;raf40;RAF24Δ10;raf18;raf20;raf35;RAF42Δ6
OK130-null: raf16;raf40;RAF24Δ10;raf24;raf18;raf20;raf35;RAF42Δ6;raf42
c
RAF40 228ins31bp resulted in a frameshift at codon 76.
RAF24 del41-70 resulted in a deletion from codon 15 to 24.
RAF18 del522-539 and 762insT resulted in a frameshift at codon 254.
RAF20 del542-1926 resulted in 1385 bp deletion.
RAF35 2700insT resulted in a frameshift at codon 900.
RAF42 del2860-2887 resulted in a deletion from codon 923 to 928 in the kinase domain.
d
Col-0
OK130-weak
0 2.5 5 15 30 60 0 2.5 5 15 30 60 min
(kDa)
130-
100-
70-
55-
45-
35-
-OK130
-OK100
*
SnRK2.2/3/6
SnRK2.1/4/5/9/10
e
RAF24 267insT resulted in a frameshift at codon 89.
RAF42 270insA resulted in an early termination at codon 91.
f
Col-0
OK130-weak
OK130-null
OK130-null-2
(kDa)
130-
100-
70-
55-
45-
35-
-OK130
-OK100
*
SnRK2.2/3/6
SnRK2.1/4/5/9/10
control
ABA
Mannitol
g
WT
OK130-weak
OK130-null
h
WT
OK130-null

Supplementary Fig. 2 Identification of OKs by biochemistry and genetics. **a** In-gel kinase assay showing the SnRK2, OK^{100}, and OK^{130} activities before (left panel) and after (right panel) mannitol treatments in wild type and single mutants of RAFs. Image is representative of two independent experiments. **b** Mutations of B4 group Raf-like kinase genes in *OK¹³⁰-weak* and *OK¹³⁰-null* alleles. The mutations in *OK¹³⁰*-weak are indicated in blue, and additional mutations in *OK¹³⁰-null* mutant are indicated in red. Ins, insertion. Del, deletion. **c** Sequencing results showing the mutations in *RAFs* in the *OK¹³⁰-weak* mutant. **d** In-gel kinase assay showing the SnRK2, OK^{100}, and OK^{130} activities in the wild type and *OK¹³⁰-weak* allele after the indicated time of mannitol treatment. Image is representative of two independent experiments. **e** Sequencing results showing the additional mutation in *RAF24* and *RAF42* in *OK¹³⁰-null* mutant. **f** In-gel kinase assay showing the SnRK2, OK^{100}, and OK^{130} activities in the wild type, *OK¹³⁰-weak, OK¹³⁰-null,* and a second null allele of OK^{130} (*OK¹³⁰-null-2*) after 15 min ABA or mannitol treatment. Image is representative of two independent experiments. **g** Photographs of inflorescences from the wild type, *OK¹³⁰-weak*, and *OK¹³⁰-null*. **h** Photographs of silique from wild type and *OK¹³⁰-null* allele. Source data are provided as Source Data file.

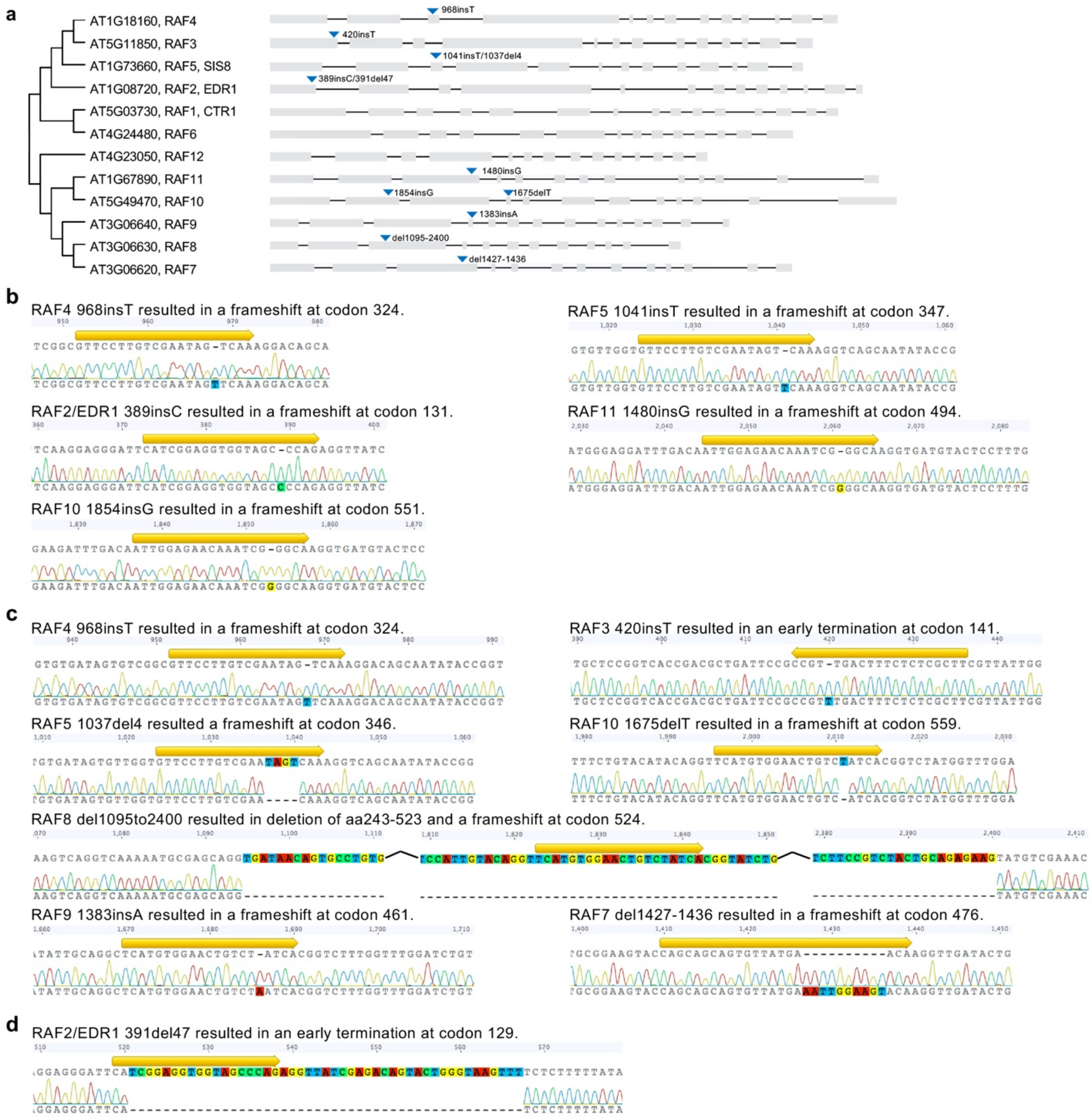

Supplementary Fig. 3 Screening of B2 and B3 RAF high-order mutants. **a** Mutations of RAF genes in the high-order mutants. The mutations are indicated by blue arrows. Ins, insertion. Del, deletion. **b** Sequencing results showing the mutations in *RAF2/EDR1*, *RAF4, RAF5, RAF10,* and *RAF11* in the *OK*^{100}*-quin* mutant. **c** Sequencing results showing the mutations in *RAF3, RAF4, RAF5, RAF7, RAF8, RAF9,* and *RAF10* in the *OK-quatdec* mutant. **d** Sequencing results showing an additional mutation in *RAF2/EDR1* in the *OK-quindec* mutant.

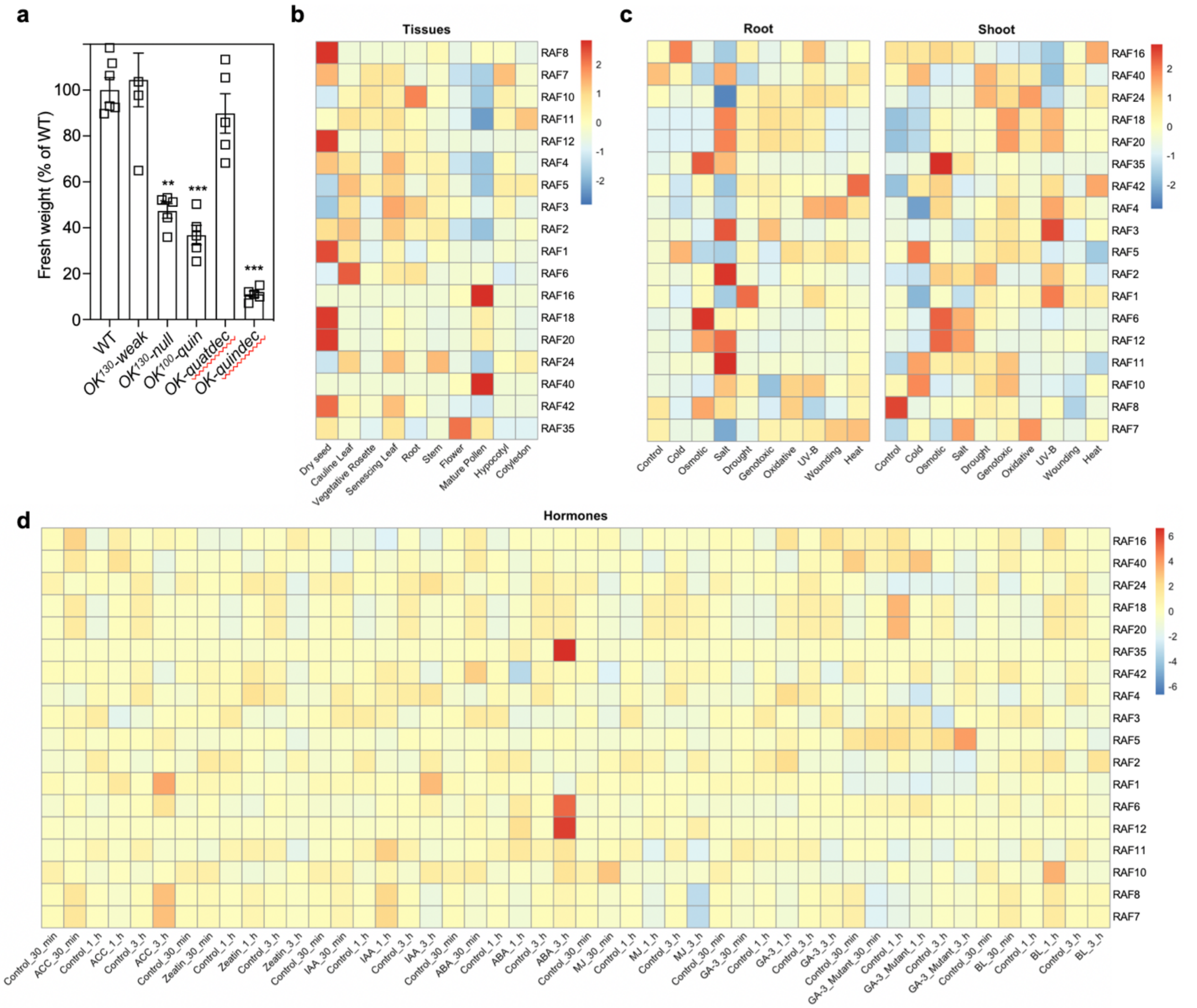

Supplementary Fig. 4 Expression pattern and localization of RAFs. **a** The fresh weight of seedlings after 4 weeks of growth in the soil. Error bars, SEM (n = 5). Two-tailed paired *t*-tests, **, $p < 0.01$, ***, $p < 0.001$. **b** Heat map showing relative abundance of *RAF* transcripts in different tissues. **c** Heat map showing relative abundance of *RAF* transcripts upon different stress treatments in root (left panel) or shoot (right panel). **d** Heat map showing relative abundance of *RAF* transcripts in control and hormone-treated seedlings. The expression of *RAFs* were obtained from the Arabidopsis eFP browser web site http://bar.utoronto.ca/efp/cgi-bin/efpWeb.cgi. Source data are provided as Source Data file.

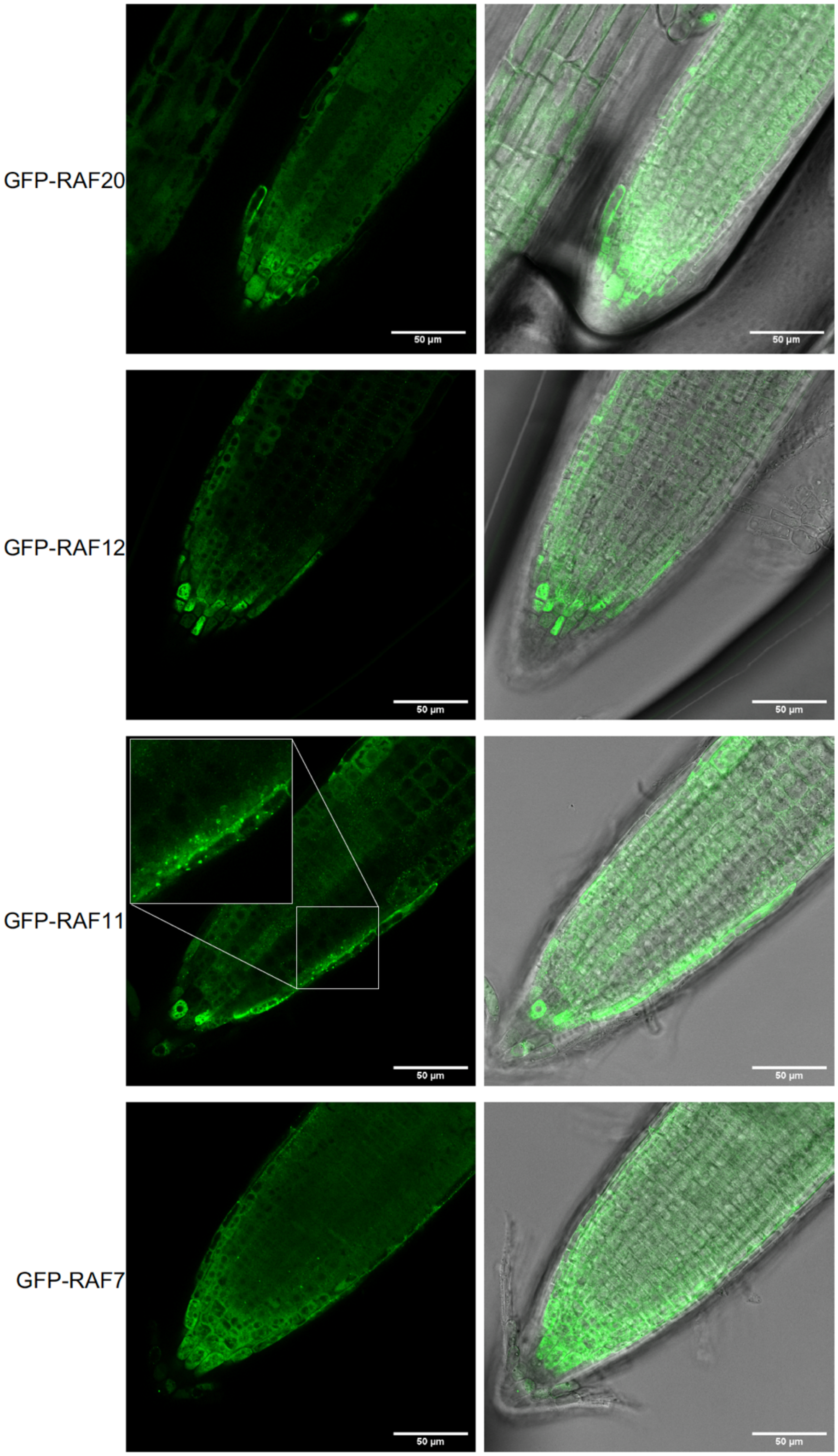

Supplementary Fig. 5 Subcellular localization of RAFs. GFP fluorescence showing the subcellular localization of GFP-RAF20, GFP-RAF12, GFP-RAF11, GFP-RAF7 in the *p35S-GFP-RAF* fusion transgenic plants.

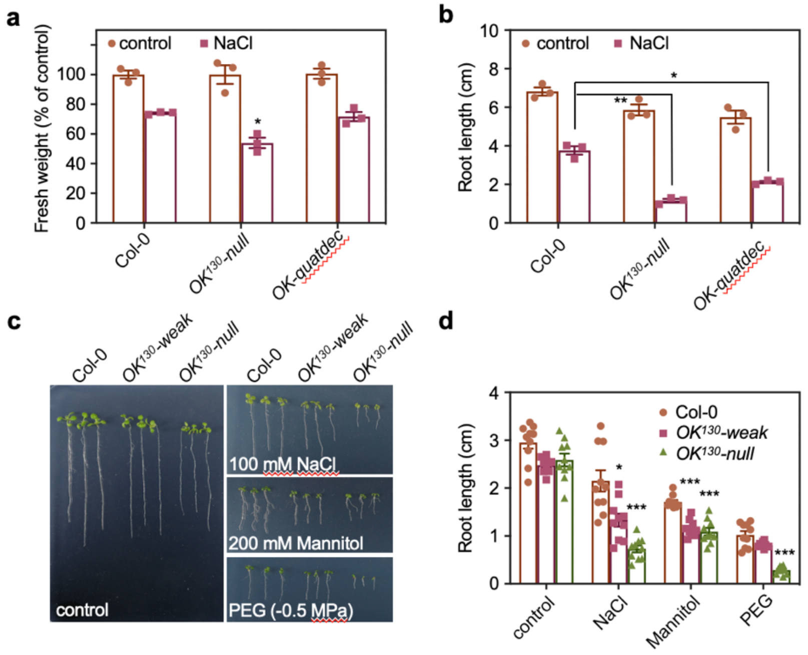

Supplementary Fig. 6 RAF high-order mutants hypersensitive to osmotic stresses. **a** The fresh weight of seedlings 7 days after transfer to and growth on medium containing 100 mM NaCl. Error bars, SEM (n = 3). Two-tailed paired *t*-tests, *, $p = 0.037$. **b** The root length of seedlings 7 days after transfer to and growth on medium containing 100 mM NaCl. Error bars, SEM (n = 3). Two-tailed paired *t*-tests, * $p = 0.025$, **, $p = 0.002$. **c** Photographs of seedlings after 7 days of germination and growth on 1/2 MS medium containing NaCl, mannitol, or PEG. **d** The root length of seedlings after 7 days of germination and growth on 1/2 MS medium containing NaCl, mannitol, or PEG. Error bars, SEM (n = 11). Two-tailed paired *t*-tests, * $p = 0.033$, ***, $p < 0.001$. Source data are provided as Source Data file.

a

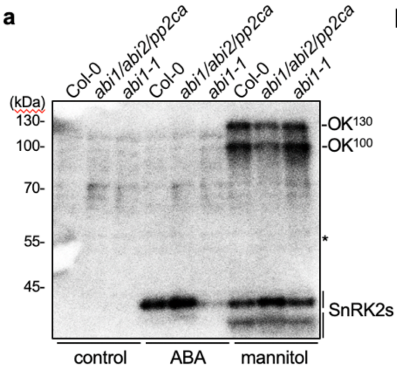

b

Bait	RAF40-GFP		RAF20-GFP		RAF35-GFP		GFP	
	Score	Peptides	Score	Peptides	Score	Peptides	Score	Peptides
GFP	79	5	323	6	323	11	323	11
Bait	323	21	323	21	323	58	-	-
SnRK2.10	62	4	-	N.D.	12.8	2	-	N.D.
SnRK2.9	62	4	-	N.D.	-	N.D.	-	N.D.
SnRK2.4	62	4	-	N.D.	12.8	2	-	N.D.
SnRK2.5	-	N.D.	-	N.D.	12.8	2	-	N.D.
SnRK2.2	62	4	7.9	2	-	N.D.	-	N.D.
SnRK2.1	6.7	1	-	N.D.	12.8	2	-	N.D.

c

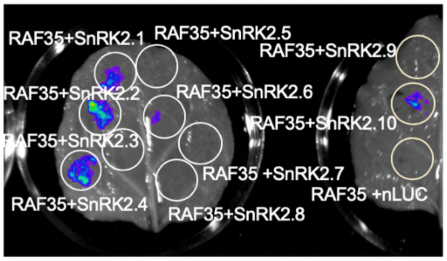

d

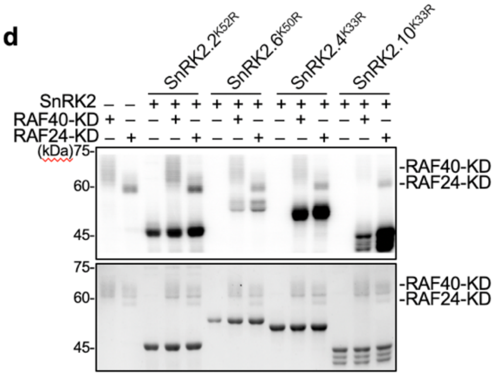

Supplementary Fig. 7 RAFs interact with and phosphorylate SnRK2s. **a** In-gel kinase assay showing the SnRK2, OK^{100}, and OK^{130} activities after ABA or mannitol treatments in wild type, *abi1/abi2/pp2ca,* and *abi1-1* mutants. Image is representative of two independent experiments. **b** Immunoprecipitation-mass spectrometry (IP-MS) result showing peptides from SnRK2s that co-immunoprecipitated with GFP-RAF40, GFP-RAF20, or GFP-RAF35, but not with empty GFP. **c** RAF35 interacts with SnRK2s in split LUC complementation assay in *Nicotiana benthamiana* leaves. The RAF35-cLUC and nLUC combination was used as a negative control. **d** RAF40-KD and RAF24-KD phosphorylates SnRK2s *in vitro*. Recombinant RAF40-KD and RAF24-KD were used to phosphorylate recombinant SnRK2s in the presence of [γ-^{32}p]ATP. Autoradiograph (upper) and Coomassie staining (bottom) show phosphorylation and loading of purified RAF-KD and SnRK2s, respectively. Source data are provided as Source Data file.

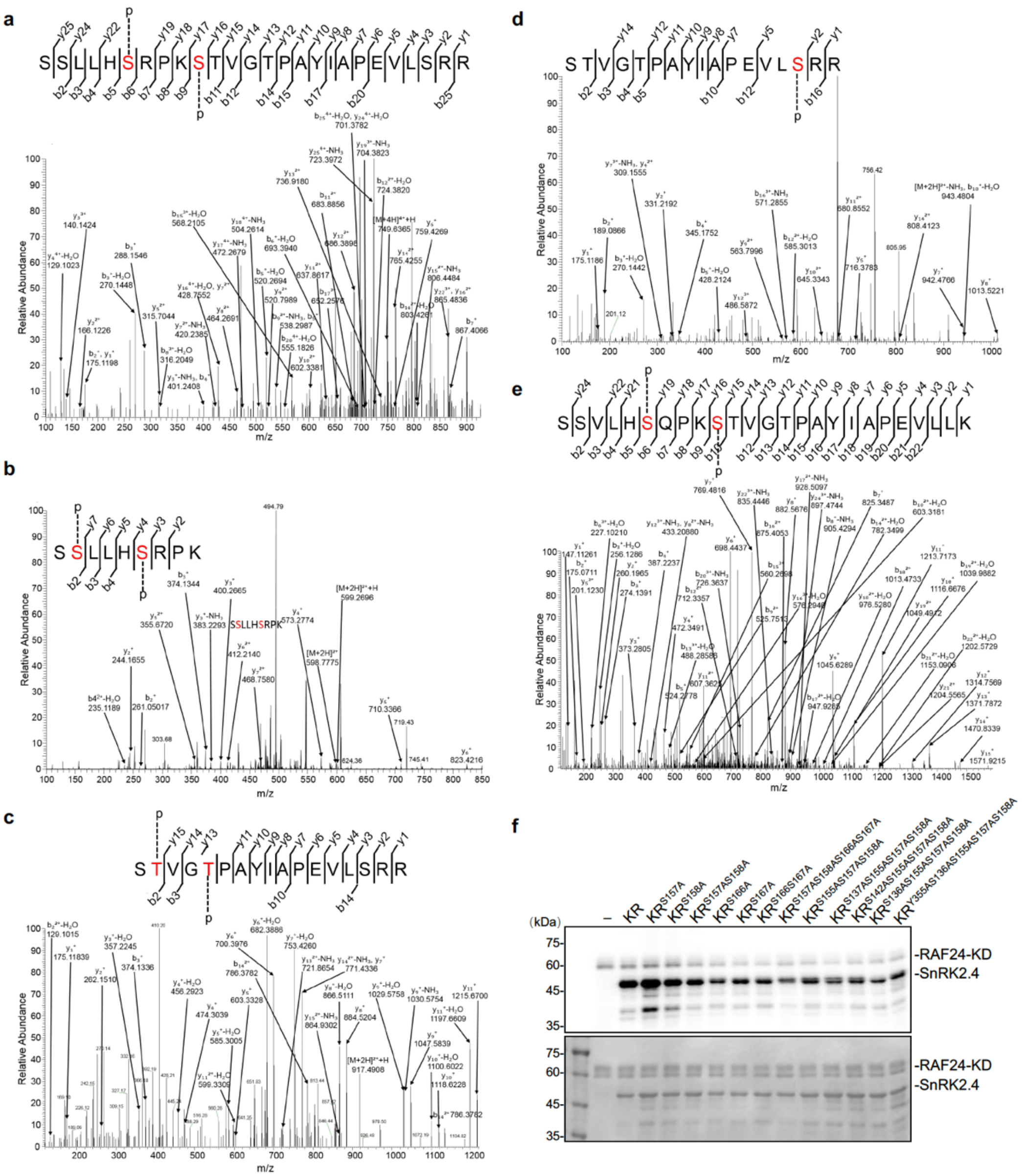

Supplementary Fig. 8 Putative RAF target sites identified in the activation loop of SnRK2.4 and SnRK2.6. **a** The MS/MS spectrum showing that the ^{18}O-phosphopeptide SSLLHSRPKSTVGTPAYIAPEVLSRR contains the phosphoserines Ser162 and Ser166 in SnRK2.4. **b** The MS/MS spectrum showing that the ^{18}O-phosphopeptide SSLLHSRPK contains the phosphoserines Ser158 and Ser162 in SnRK2.4. **c** The MS/MS spectrum showing that the ^{18}O-phosphopeptide STVGTPAYIAPEVLSRR contains the phosphothreonines Thr167 and Thr170 in SnRK2.4. **d** The MS/MS spectrum showing that the ^{18}O-phosphopeptide STVGTPAYIAPEVLSRR contains the phosphoserine Ser180 in SnRK2.4. **e** The MS/MS spectrum showing that the ^{18}O-phosphopeptide SSVLHSQPKSTVGTPAYIAPEVLLK contains the phosphoserines Ser171 and Ser175 in SnRK2.6. **f** Phosphorylation of SnRK2.4^{K33R}(KR) and phosphosite-mutated SnRK2.4^{K33R} by recombinant RAF24-KD. Autoradiograph (upper) and Coomassie staining (bottom) show phosphorylation and loading of purified RAF24-KD and SnRK2.4, respectively. Source data are provided as Source Data file.

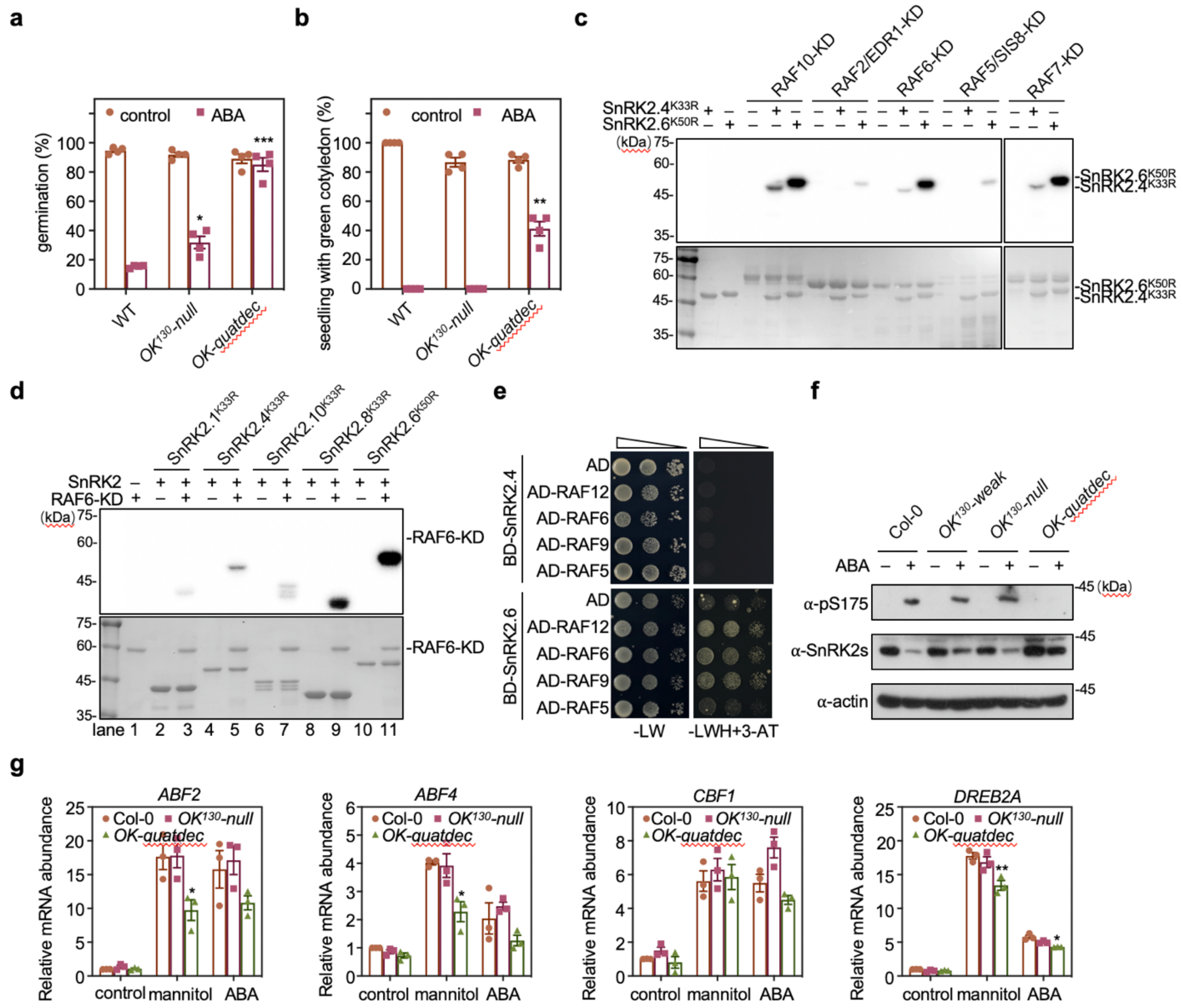

Supplementary Fig. 9 B2 and B3 subgroup RAFs mediate ABA signaling by phosphorylating SnRK2s. **a** The germination rate of wild type, *OK^{130}-null* mutant, and *OK-quatdec* mutant on 1/2 MS medium with or without ABA. Error bars, SEM (n = 4). Two-tailed paired *t*-tests, *, p = 0.017, ***, p = 0.00035. **b** The percentage of seedlings with green cotyledon in wild type, *OK^{130}-null* mutant, and *OK-quatdec* mutant on 1/2 MS medium with or without ABA. Error bars, SEM (n = 4). Two-tailed paired *t*-tests, **, p = 0.0017. **c** RAF-KDs phosphorylate SnRK2.4 and SnRK2.6 *in vitro*. **d** RAF6-KD phosphorylates SnRK2s *in vitro*. Autoradiograph (upper) and Coomassie staining (bottom) show phosphorylation and loading of purified RAF6-KD and SnRK2s, respectively. **e** SnRK2.6 but not SnRK2.4 interacts with some B2 and B3 RAFs in a yeast two-hybrid assay. **f** The ABA-induced phosphorylation of the conserved serine corresponding to Ser175 in SnRK2.6 depends on RAFs. **g** Expression of ABA responsive transcription factors in wild type, *OK^{130}-null*, and *OK-quatdec* seedlings after 6 hours of mannitol or ABA treatment. Error bars, SEM (n = 3). Two-tailed paired *t*-tests, *, $p < 0.05$, **, p <0.01. Source data are provided as Source Data file.

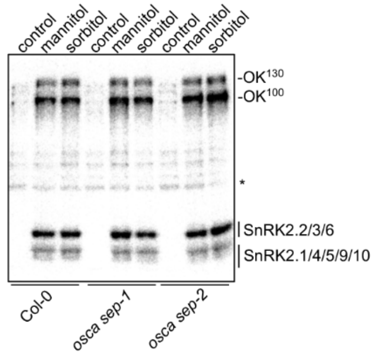

Supplementary Fig. 10 Mutation of OSCA does not affect the RAF and SnRK2 activation. In-gel kinase assay showing the SnRK2, OK^{100}, and OK^{130} activities in mannitol- and sorbitol-treated wild type and *osca-sep* mutants. Image is representative of two independent experiments. Source data are provided as Source Data file.

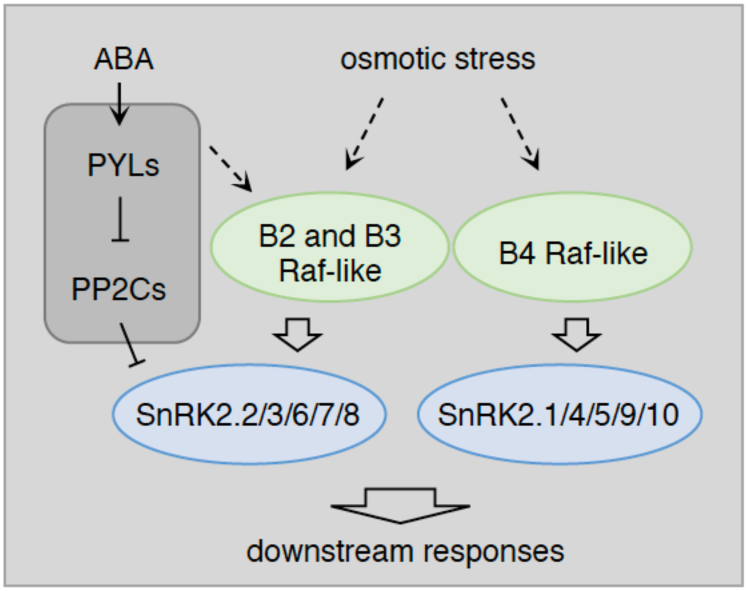

Supplementary Fig. 11 Model showing that RAFs mediate early osmotic stress and ABA signaling by phosphorylating SnRK2s.

Supplementary Table 1. The guide RNAs used for genome editing and their target sequences.

The *gRNAs* used in *pCambia 1300-6RAFs*

name	gRNA sequence	target gene
gRNA-1	AGGGGTATGATAGTGCTGCT	RAF40/AT3G24715
gRNA-2	GACCCTAGAGATGAGGGACT	RAF24/AT2G35050
gRNA-3	AATGAGATGGAAAACCGTGG	RAF18/AT1G16270<br>RAF20/AT1G79570
gRNA-4	AGCTTTGAATCACCTGGACG	RAF18/AT1G16270<br>RAF20/AT1G79570
gRNA-5	GATGAGGATCTTGATGCTT	RAF35/AT5G57610<br>RAF42/AT3G46920
gRNA-6	TTTGGGATGGAGTATTTGCA	RAF35/AT5G57610<br>RAF42/AT3G46920

The *gRNAs* used in *pCambia 2300 2RAFs*

name	gRNA sequence	target gene
gRNA-7	AATGTGTATGGTGAGCACAG	RAF24/AT2G35050
gRNA-8	AACTACACAAGAACTTCGCA	RAF24/AT2G35050
gRNA-9	ATAATCCCTAGACCAAGTGA	RAF42/AT3G46920
gRNA-10	CCGGCCAATCTTTCAGCTTC	RAF42/AT3G46920

The *gRNAs* used in *pCambia 2300 5RAFs*

name	gRNA sequence	target gene
gRNA-11	TGTAGTCCAGCTGCATCAGC	RAF4/AT1G18160<br>RAF5/AT1G73660
gRNA-12	GTTCCTTGTCGAATAGTCAA	RAF4/AT1G18160<br>RAF5/AT1G73660
gRNA-13	CGTCTCAGTGACTGACGAAG	RAF2/AT1G08720
gRNA-14	CATCGGAGGTGGTAGCCCAG	RAF2/AT1G08720
gRNA-15	CAGTGTTAAGTCTGAAAGCC	RAF11/AT1G67890<br>RAF10/AT5G49470
gRNA-16	ATTGGAGAACAAATCGGGCA	RAF11/AT1G67890<br>RAF10/AT5G49470

The *gRNAs* used in *pCambia 2300 11RAFs*

name	gRNA sequence	target gene
gRNA-12	GTTCCTTGTCGAATAGTCAA	RAF3/AT1G18160<br>RAF5/AT1G73660
gRNA-17	CAGCAGCAGTGTTATGAACA	RAF7/AT3G06620<br>RAF11/AT1G67890<br>RAF10/AT5G49470
gRNA-18	AAGCGAGAGAAAGTCAACGG	RAF3/AT5G11850
gRNA-19	CAGAAGCTCTCTCTCCTCAT	RAF6/AT4G24480
gRNA-20	TCATGTGGAACTGTCTATCA	RAF8/AT3G06630<br>RAF7/AT3G06620<br>RAF11/AT1G67890<br>RAF10/AT5G49470
gRNA-21	TGGAAAGGGAATGAGCAGGA	RAF9/AT3G06640<br>RAF7/AT3G06620
gRNA-14	CATCGGAGGTGGTAGCCCAG	RAF2/AT1G08720
gRNA-22	GACGGGATTGGAGACGGACA	RAF12/AT4G23050
