# Supplementary material for: A RAF-SnRK2 kinase cascade mediates early osmotic stress signaling in higher plants
Source: Nat Commun. 2020 Jan 30;11:613. doi: 10.1038/s41467-020-14477-9 (PMC6992735; doi:10.1038/s41467-020-14477-9)
Supplement: Supplementary file 3 — Description of Additional Supplementary Files [file 41467_2020_14477_MOESM3_ESM.pdf]

Description of Additional Supplementary Files

File Name: Supplementary Data 1
Description: The mannitol-perturbed phosphosites from Col-0 Arabidopsis.

File Name: Supplementary Data 2
Description: The mannitol-perturbed phosphosites from snrk2-dec mutant Arabidopsis.

File Name: Supplementary Data 3
Description: All the identified kinase phosphosites from Col-0 and snrk2-dec mutant Arabidopsis.

File Name: Supplementary Data 4
Description: The mannitol-induced kinase phosphosites from Col-0 Arabidopsis.

File Name: Supplementary Data 5
Description: The mannitol-induced kinase phosphosites from snrk2-dec mutant Arabidopsis.

File Name: Supplementary Data 6
Description: The 18O labeled putative RAF target peptides in SnRK2.4 and SnRK2.6.

File Name: Supplementary Data 7
Description: Primers used in this study.
